# What Is New in Psychiatry and Psychopharmacology

**DOI:** 10.3390/life15020220

**Published:** 2025-02-01

**Authors:** Donatella Marazziti

**Affiliations:** Department of Clinical and Experimental Medicine, Section of Psychiatry, University of Pisa, Via Roma 57, 56100 Pisa, Italy; dmarazzi@psico.med.unipi.it

Psychiatry and psychopharmacology are rapidly evolving due to the developments in different domains and the constantly increasing demands for psychiatric care worldwide amongst children, adolescents, adults, and elderly populations, who all require specific and tailored management and treatment strategies [1]. It is now evident that psychiatric disorders may often begin in childhood or adolescence and greatly impair subsequent development and adjustment [2,3]. As such, they require both early detection and a personalized therapeutic approach, taking into account individual vulnerabilities as well as environmental (that is to say, social and physical) factors. In our opinion, the notion that the complexity of psychiatric disorders results from what we call “wicked relationships” amongst different factors is a major achievement and a cornerstone of the research of the last decade [4,5]. In any case, it is fundamental to increase and consistently improve the knowledge of psychiatry in both medical and in specialty schools so that even a physician can promptly diagnose a given psychopathological condition.

Undoubtably, clinical observations have become more accurate and more responsive to the therapeutic demands and challenges deriving from the changing world and the novel problems it creates in this field. For instance, at present we have to cope with a series of unpredictable dramatic events, such as war in different parts of the world, terror attacks, economic recession, famine, climate change leading to extreme meteorological events that spoil territories and force people to migrate, the consequences of the COVID-19 pandemic, and pathological Internet use. It can be predicted that these events will further increase the number of individuals seeking psychiatric help and requesting the appropriate pharmacological management of psychiatric disorders [6]. Again, there is a major need for more targeted and effective drugs and more focused psychological interventions to resolve the high percentage of non-response amongst patients with different disorders. Unfortunately, the demand for innovation may clash with current economic constraints, with limited resources being available for clinical practice and research following a shift towards the management of the COVID-19 pandemic.

The development of novel drugs depends on research, which should be promoted and move beyond classical paradigms. However, innovative hypotheses require courage, a bit of bravery, and creativity, as well as the awareness that only collaboration between researchers, clinicians, government organizations, and pharmaceutical companies can lay the foundation for a better future.

I consider it an honor and a privilege to collaborate with the MDPI journal *Life* to organize this Special Issue entitled “What Is New in Psychiatry and Psychopharmacology”, which includes various contributions highlighting the current achievements and future developments in these domains.
